# Segmented whole body haemodynamic responses to a high calorie meal - a novel MR approach

**DOI:** 10.1186/1532-429X-17-S1-P31

**Published:** 2015-02-03

**Authors:** Jakob A Hauser, Vivek Muthurangu, Jennifer Steeden, Andrew Taylor, Alexander Jones

**Affiliations:** 1 Institute of Cardiovascular Science, University College London, London, UK

## Background

Metabolic and endocrine responses to food intake vary, altering risk of obesity and cardiovascular disease. The importance of variations in haemodynamic response is unknown. Technical difficulties and lack of an accurate, reliable, non-invasive means to measure small vessel blood flow have prevented significant advances. We tested whether novel MR flow sequences could overcome this obstacle.

## Methods

In 9 healthy young adults, we used accelerated MR sequences to quantify flow throughout the body at rest and every 10 minutes for an hour, after a high calorie liquid meal (300ml of double cream + 89g of maltose; 2350 kcal equivalent; carbohydrate content equivalent to glucose tolerance test). High spatiotemporal resolution gated spiral phase-contrast MR was used in large vessels (aorta [ascending, descending, bifurcation]) and RR interval averaged golden angle spiral phase contrast MR was used in small vessels (carotid, vertebral, renal, and superior mesenteric [SMA] arteries, coeliac trunk and portal vein) due to their small diameter precluding measurement with conventional MR sequences. Each subject then underwent the same protocol, drinking equivalent volumes of water. Changes over time and comparisons between meal and water responses were assessed using multilevel linear regression.

## Results

Despite the high calorie load, there was no vomiting and no significant nausea. Meal ingestion increased cardiac output substantially (~1.5 litres), whereas water ingestion had little effect. Stroke volume and heart rate increases were responsible, with heart rate becoming more dominant over time. There was no significant flow increase to the head, kidneys or legs. Intestinal blood flow (coeliac trunk, SMA, portal vein) was responsible for the increase in cardiac output in the first hour after the meal. The figure shows results from selected multilevel models, adjusted for sex, age and BMI. Aortic flow, stroke volume and heart rate changes compared to rest were significantly different between meal and water ingestions. This was also true for SMA flow but not cerebral, renal or distal aortic flow.

**Figure 1:**
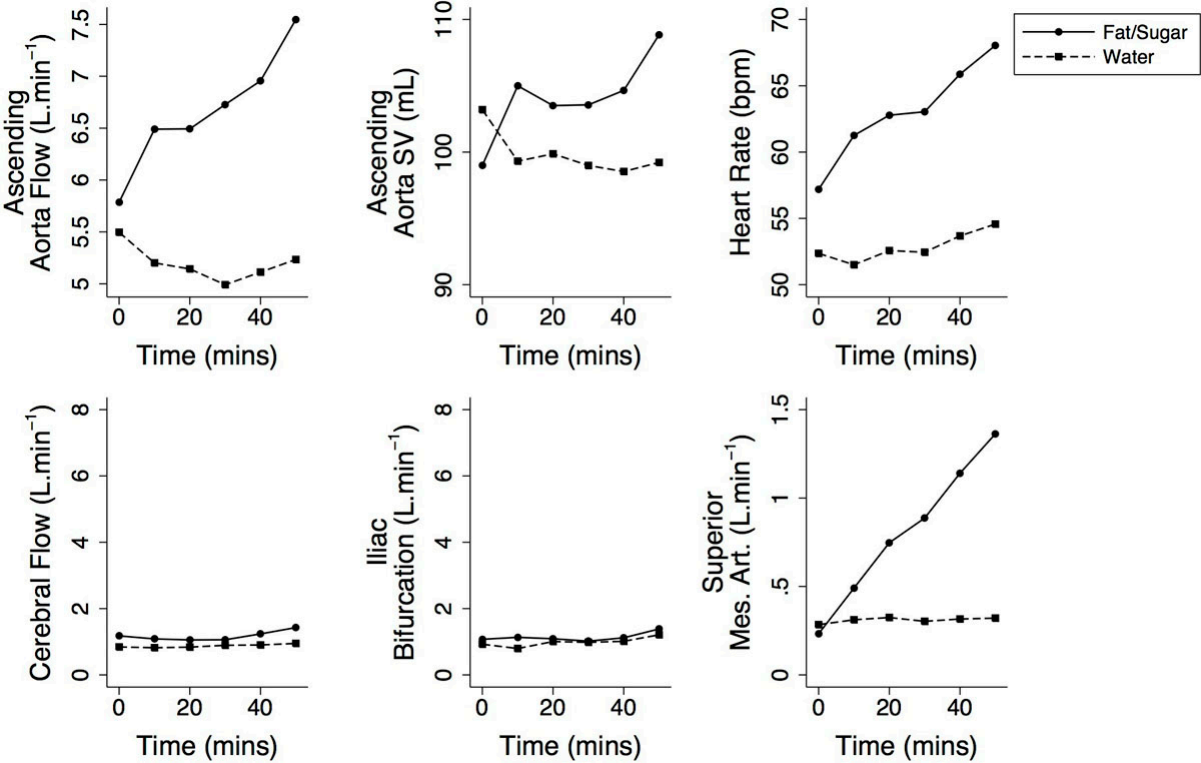


## Conclusions

This is the first comprehensive characterisation of regional blood flow responses to a meal using a non-invasive technique and a novel, MR-compatible high-calorie meal. The protocol was well tolerated and showed clear differences between groups, demonstrating that the haemodynamic response was not due to a volume load. This technique may be useful for testing whether variations in intestinal blood flow impact on the risk of metabolic disorders such as obesity.

## Funding

We acknowledge the support of the British Heart Foundation, UK National Institute of Health Research (NIHR) and the University College London / UCLH Biomedical Research Centre. JH is funded by an EU FP7 EME grant (MD-PAEDIGREE).

